# Is vegetarianism related to anxiety and depression? A cross-sectional survey in a French sample

**DOI:** 10.1186/s41043-022-00300-2

**Published:** 2022-05-09

**Authors:** Laurent Bègue, Rebecca Shankland

**Affiliations:** 1 LIP/PC2S, University Grenoble Alpes, Grenoble, France; 2 University Lyon 2 Lumière, DIPHE, Lyon, France

## Abstract

**Objective:**

The relationship between vegetarianism and mental health is controversial. The aim of the present study is to examine the cross-sectional association between anxiety, depression, and vegetarianism in a French sample while controlling for potential confounders.

**Design:**

Self-reported questionnaire data were obtained from a large cross-sectional sample.

**Participants and setting:**

We included an online sample of 6578 participants aged 18–90, 70.8% females.

**Results:**

Analyses of variance with age, gender, body mass index (BMI), educational level, monthly income, and city size as covariates showed that vegetarians and non-vegetarians did not appear to have significantly different levels of anxiety or depression.

**Conclusion:**

Our findings do not suggest a link between plant-based diet and anxiety or depression, either before or after adjustment for relevant factors.

## Background

It has been estimated that there are about 1.5 billion vegetarians worldwide. After a long history in Asian cultures, the popularity of plant-based diets is currently increasing in the Western world. According to recent polls, approximately 10% people exclude meat from their diets in New Zealand, Australia, and Israel [1]. They represent 8% of the population in Canada [2] and UK [3], 5% in the USA [4], Germany [5], and France [3], and 3% in Spain [3]. In France, a recent rise in vegetarian consumption has been observed alongside a 9% decrease in meat consumption in recent years [3]. Three main motivations have been shown to underlie vegetarianism: sustainability issues [6], health benefits [7, 8], and animal welfare [9]. Regarding health benefits, it has been widely documented that excessive meat consumption increases the risks of cancer [10, 11], heart diseases [12], strokes [13–15], type 2 diabetes [14, 16, 17], and obesity [7, 18]. The health benefits of eating less or no meat have been underlined by the Academy of Nutrition and Dietetics [19] and may be explained by a higher intake of fiber, polyunsaturated fats, vitamin C, bioactive molecules, and a lower consumption of saturated fat [20, 21]. However, the consequences of vegetarianism on a person’s mental health remain controversial. Despite several studies showing that vegetarians generally follow diets that are closer to public health guidelines than those of omnivores [22], some studies have also suggested that potential nutritional deficits among vegetarians in relation to vitamin B12, long-chain n-3 fatty acids, folates, and/or zinc [23–25] may lead to adverse psychological outcomes.

Anxiety and depression both count among these adverse psychological outcomes. Anxiety is a psychological state characterized by feelings of tension, recurring intrusive thoughts or concerns, and physical changes such as increased blood pressure. Depression is a negative affective state, ranging from unhappiness and discontent to an extreme feeling of sadness that interferes with daily life, which is associated with a lack of energy or motivation, difficulty in concentrating or making decisions, and withdrawal from social activities. A link between vegetarianism or low meat consumption and anxiety and/or depression has been observed in several prior studies [22–27]. For example, in a recent German study, the percentage of participants who screened positive for potential cases of depression and anxiety was 5.3% in omnivores and 8.5% in vegetarians or vegans [25]. In the largest existing single study [28], which involved a sample of 90,380 French participants, depressive symptoms were associated with exclusion of meat and fish and a lacto-ovo-vegetarian diet (exclusion of meat, fish, eggs, and dairy products) in a multivariate analysis controlling for an extended number of relevant covariates (e.g., age, sex, income, education level, physical activity, smoking, and alcohol intake). In that sample, 28.4% of meat-abstainers showed symptoms of depression, compared to only 16.2% of meat consumers. However, the observed association between a vegetarian diet and depression only represented a particular case of a broader association between depressive symptoms and food exclusion, regardless of food type. It therefore seemed non-specific to the exclusion of animal food but, rather, to underlie food avoidance in general. The authors also noted that depressive symptoms were not associated with a vegetarian diet among individuals with high legume intake.

A few other studies carried out in general population samples have not confirmed an increased risk of depression or anxiety among vegetarians [29–31]. In a cross-sectional and longitudinal culturally diverse and representative sample, vegetarianism was found to be unrelated to mental health in the USA, Russia, and Germany and was only slightly predictive of depression and anxiety among Chinese students [32]. In another study with 12,905 participants in Germany and 15,532 Australians, the authors concluded that the effect of diet on subjective well-being was either nonexistent or negligible [31]. No relationship has been found in specific populations like endurance runners [33] or people from developing countries either [34].

Finally, a few studies have found lower levels of anxiety and depression among people adopting a vegetarian diet [29, 34, 35]. In the only randomized controlled trial currently available, a large multicenter nutrition intervention found that a vegan diet (without any animal products) appeared inversely related to depression and anxiety [35]. However, as was acknowledged by the authors of that study, the control group did not receive any intervention, and the mere presence of an intervention in the nutrition intervention group could have accounted for the observed effects.

The contradictory results observed in the aforementioned studies may be the consequence of many factors such as research design, sampling methodology, the nature of the nutritional measures used (self-reported or actual intake), the validity of outcome measures, and the lack of statistical controls on relevant variables. Three recent systematic reviews have provided results based on aggregated independent research studies [19, 36, 37]. In the first one, a large comprehensive synthesis based on eighteen studies comprising a total of 160,257 participants, it was concluded that meat-abstention was related to higher rates of risk of depression, anxiety, and related symptoms [38]. This systematic review showed that seven out of 14 studies had found a higher prevalence or risk in participants who avoided meat, while three found no differences between groups, two showed mixed results, and the remaining two found a higher risk among meat consumers. Moreover, the authors estimated the quality of the included studies and observed that the four more rigorous studies indicated a link between avoidance of meat and risk of depression, anxiety, and related symptoms. The second meta-analysis on vegetarianism and mental health was quantitative, an approach which is supposed to provide higher-quality results [37]. It included thirteen studies (total *N* = 147,964) published between 2005 and 2020, with samples from Europe, Asia, and the USA. The results reported that no association was found between adherence to a vegetarian diet and the prevalence of depression and anxiety [36]. The third available meta-analysis included thirteen studies with a total of 17,809 participants and concluded that there was no statistically significant difference between vegetarian or vegan diets and omnivores regarding the incidence of continuous measures of depression, stress, and well-being. Moreover, individuals who followed vegetarian and vegan diets had lower levels of anxiety. However, when depression was analyzed as a categorical variable, vegetarians and vegans had a higher risk of depression [19]. It has been proposed that the contrasting conclusions of these three meta-analyses might be attributable to differences in their reviewing methodologies [37].

Given the still uncertain relationship between vegetarianism and depression, we aimed to determine whether vegetarianism was related to depressive symptomatology and happiness in a large community sample in France. In several studies, education, income, and city size were associated with meat consumption [39–41]. We therefore measured and controlled for these relevant covariates. We also measured body mass index, which is considered a relevant measure in studies on vegetarian diet and health [42–45], and was also included as a measure.

In summary, the aim of the present research was to advance the understanding of the possible link between vegetarianism, anxiety, and depression. To that end, we relied on a large community sample enabling the inclusion and the statistical control of relevant covariates.

## Methods

### Setting and sample

There was no a priori defined sample size. Following a public campaign in the national media, the study’s participants had previously freely registered on a Web site promoting meat and fish substitution once a week. The campaign, called “Green Monday” (or “Lundi Vert” in French), officially started in January 2019 with a massive press release of a petition with 500 public figures’ signatures, including those of well-known artists, sportsmen, politicians, scientists, and NGOs, calling on consumers to change their eating habits and avoid eating meat and fish every Monday throughout 2019 for environmental, health, and animal welfare reasons [46]. Most French news Web sites, as well as the country’s public television and radio broadcasts, publicized the campaign, and large 3 × 4 posters were also displayed in 60 subway stations in Paris. Participants in the present study completed an online survey in December 2019, eleven months after the launch of the campaign. The total sample size was 9993 participants. Those who did not indicate their gender (*n* = 1371) or who were not eligible to participate due to being aged below 18 (*n* = 612), or did not reply in any other question were deleted. The final sample therefore included 6578 participants (70.1% females), aged 18–90 years old.

### Measures

#### Covariates

Due to being generally related to mental health and/or food habits, the following variables were selected as covariates: age, gender, educational level, monthly income, and city size. We also included participants’ BMI [47], which was calculated from their self-reported weight and height (weight divided by height squared; kg/m2). The participants were asked to report their educational level (from 1 = lowest level below baccalaureate, to 7 = doctorate or other degrees), their current net monthly income (from 1 = less than 1000 euros to 6 = more than 4500 euros), and the size of their city of residence (from 1 = less than 10,000 inhabitants to 6 = more than 400,000 inhabitants).

#### Main variables

Due to time constraints, short measures were preferred over longer and/or extended multidimensional measures. We relied on the GAD-2 scale [48] to evaluate anxiety (*M* = 2.30, SD = 2.00, Cronbach’s alpha = 0.81, example item: “In the last two weeks, how often have you been bothered by the following problems: feeling nervous, anxious, or on the edge”). The PGQ-2 scale [49] was used to estimate depression (*M* = 1.72, SD = 1.50, Cronbach’s alpha = 0.80, example item “In the last two weeks, how often have you been bothered by the following problems: Feeling down, depressed of hopeless”). Response options for both measures ranged from 1 = not at all, to 5 = every day. Regarding food exclusion habits, the participants were asked which foods they never (or almost never) consumed, from a list including meat (beef, chicken, pork, and others), fish, milk, and dairy products (cheese, yoghurts, etc.), or other foods. We observed that 4.2% of participants excluded eggs, 9.2% excluded dairy products, 17.7% excluded fish, and 28.2% excluded meat. These observations suggest that the sample was not representative of food habits in France, where vegetarianism is estimated between 2 and 5% of general population. While these features would raise an issue if the aim of the study was to provide absolute values regarding food habits in France, the fact that vegetarians were oversampled enabled us to ensure a comparison with enough statistical power.

#### Statistical methods

Participants with any missing values were excluded from the analysis. Two analyses of covariance were performed to compare the anxiety and depression scores of the vegetarian and the non-vegetarian group controlling for relevant factors (see below). Regarding the dichotomization of the variable, participants who declared that they avoided both meat and fish (12.8%, *N* = 839) were classified in the vegetarian group, coded 1, which was contrasted with a category for those who ate meat, fish, or both (87.2%, *N* = 5739), coded 0. Age, gender, BMI, educational level, monthly income, and city size were all entered covariates, and anxiety (GAD-2 scale) and depression (PhQ-2 scale) were entered as dependent variables in the models.

#### Ethical aspects

All procedures performed were in accordance with the ethical standards for questionnaire studies at University Grenoble Alpes, and with the 1964 Helsinki Declaration and its later amendments or comparable ethical standards.

## Results

A univariate analysis with Bonferroni corrections (Table 1) indicated that vegetarians were not significantly different in depression or anxiety.

**Table 1:** Characteristics of the samples and univariate comparisons

	Not vegetarians (*N* = 5739)	Vegetarians (*N* = 839)	Statistical tests
Age (M. SD)	60.85 (22.06)	60.06 (21.81)	*t *(6576) = 0.96, ns
Education level (1–7) (mean, SD)	4.20 (1.35)	4.10 (1.34)	*t*_cor _(1101.04) = 2.04, ns
Income (1–6) (mean, SD)	4.20 (1.41)	3.71 (1.50)	*t*_cor _(1066.06) = 8.80, *p*. < .001
City size (1–6) (mean, SD)	1.27 (1.18)	1.01 (1.18)	*t*_cor _(1095.91) = 2.05, *p*. < .001
BMI (mean, SD)	23.35 (3.97)	22.24 (3.47)	*t*_cor_ (1184.23) = 8.47, *p*. < .001
Anxiety, GAD 2 (1–5) (mean, SD)	2.30 (1.04)	2.31 (1.04)	*t *(6576) = 0.20, ns
Depression, PHQ2 (1–5) (mean, SD)	1.72 (0.84)	1.77 (0.86)	*t *(6576) = 1.72 ns

*BMI* body mass index, *GAD2* Generalized Anxiety Disorder Scale, *PGQ scale*, Patient Health Questionnaire Scale, *NB* due to Bonferroni correction, the significance threshold is 0.005

The analysis of covariance including gender, age, city size, income, and BMI indicated that vegetarians and omnivorous were not significantly different regarding anxiety score (GAD-2 scale, *M* = 2.31, SD = 1.04 vs. *M* = 2.30, SD = 1.04, F(1,6577) = 0.43, *p* = 0.83) and depression score (PhQ-2 scale, − *M* = 1.72, SD = 0.84 vs. *M* = 1.77, SD = 0.86, F(1,6577) = 1.01, *p* = 0.31). The results of both analyses are presented in Tables 2 and 3.

**Table 2:** Analysis of covariance with anxiety (GAD-2 scale) as a dependent variable

	Type III sum of squares	ddl	Mean square	*F*	*p* value
Corrected model	72.54	7	10.36	9.56	.000
Constant	413.50	1	413.50	381.53	.000
Gender	4.85	1	4.85	4.48	.03
Age	5.29	1	5.29	4.88	.02
City size	14.72	1	14.72	13.58	.000
Educational level	15.84	1	15.84	14.62	0.30
Income	24.01	1	24.01	22.15	.000
BMI	5.48	1	5.48	5.05	.025
Vegetarianism	0.46	1	0.46	0.43	0.83
Error	7120.47	6570	1.08		
Total	42,251.34	6578			
Corrected total	7193.01	6577

**Table 3:** Analysis of covariance with depression (PHQ-2 scale) as a dependent variable

	Type III sum of squares	ddl	Mean square	*F*	*p* value
Corrected model	124.51	7	17.78	25.39	.000
Constant	194.67	1	194.67	277.94 .000	
Gender	0.78	1	0.78	1.12	0.28
Age	5.07	1	5.07	7.24	.007
City size	11.85	1	11.85	16.86	.000
Educational level	0.72	1	0.72	1.03	0.30
Income	33.33	1	30.33	43.31	.000
BMI	30.67	1	30.67	43.79	.000
Vegetarianism	0.71	1	0.71	1.01 0.31	
Error	4601.786	6570	0.700		
Total	24,365.249	6578			
Corrected total	4726.30	6577			

## Discussion

The main objective of this study was to investigate the possible associations between vegetarianism, anxiety, and depression, controlling for relevant potential confounders. Our results showed that individuals with a vegetarian diet did not present an increased risk of anxiety or depression.

Some limitations affecting this study need to be discussed. Firstly, our measures were only based on self-reported and declarative measures. The possibility therefore cannot be excluded that some participants answering that they rejected meat or fish were actually not fully abstaining from these foods, as many earlier studies have suggested [36, 38]. Moreover, in this study, some important potential covariates, such as alcohol and tobacco consumption, were not measured [50, 51], and the possibility of response bias in different age-groups was not estimated. Finally, the sample was not representative of the country’s general population, as it was reached through a specific national campaign. This specificity regarding our sample imposes a limitation on the generalization of our results, but it also represents an asset. The individual profiles of participants included in our sample were indeed more homogeneous because they all had in common the fact of having registered on a Web site to limit the consumption of meat every Monday. Consequently, vegetarians and non-vegetarians in the current study were probably more similar in many other personality and social variables, which decreased the risk of a spurious relationship between following a plant-based diet and our main measures. It has been shown that health, environmental, and ethical motives represented important motivations for vegetarianism [52]. Future studies could include an exploration of the underlying motivations for vegetarianism. Differentiating subtypes of vegetarians could also contribute to clarifying the link between this specific eating behavior and indices of mental health.

In conclusion, a vegetarian diet appears to be unrelated to anxiety or depression in this study. Further studies should investigate the generalizability of these results in a representative sample of French participants.

## Data Availability

Data are fully available on request to Laurent.Begue@univ-grenoble-alpes.fr.

## Abbreviations

BMI: Body Mass Index
GAD scale: Generalized Anxiety Disorder Scale
PGQ scale: Patient Health Questionnaire Scale
